# Heterogeneity of Metabolic Defects in Type 2 Diabetes and Its Relation to Reactive Oxygen Species and Alterations in Beta-Cell Mass

**DOI:** 10.3389/fphys.2019.00107

**Published:** 2019-02-13

**Authors:** Andris Elksnis, Mats Martinell, Olof Eriksson, Daniel Espes

**Affiliations:** ^1^Department of Medical Cell Biology, Uppsala University, Uppsala, Sweden; ^2^Department of Public Health and Caring Sciences, Uppsala University, Uppsala, Sweden; ^3^Science for Life Laboratory, Department of Medicinal Chemistry, Uppsala University, Uppsala, Sweden; ^4^Department of Medical Sciences, Uppsala University, Uppsala, Sweden

**Keywords:** type 2 diabetes, diabetes classification, oxygen stress, reactive oxygen species, beta-cell, beta-cell mass, imaging, positron emission tomography

## Abstract

Type 2 diabetes (T2D) is a complex and heterogeneous disease which affects millions of people worldwide. The classification of diabetes is at an interesting turning point and there have been several recent reports on sub-classification of T2D based on phenotypical and metabolic characteristics. An important, and perhaps so far underestimated, factor in the pathophysiology of T2D is the role of oxidative stress and reactive oxygen species (ROS). There are multiple pathways for excessive ROS formation in T2D and in addition, beta-cells have an inherent deficit in the capacity to cope with oxidative stress. ROS formation could be causal, but also contribute to a large number of the metabolic defects in T2D, including beta-cell dysfunction and loss. Currently, our knowledge on beta-cell mass is limited to autopsy studies and based on comparisons with healthy controls. The combined evidence suggests that beta-cell mass is unaltered at onset of T2D but that it declines progressively. In order to better understand the pathophysiology of T2D, to identify and evaluate novel treatments, there is a need for *in vivo* techniques able to quantify beta-cell mass. Positron emission tomography holds great potential for this purpose and can in addition map metabolic defects, including ROS activity, in specific tissue compartments. In this review, we highlight the different phenotypical features of T2D and how metabolic defects impact oxidative stress and ROS formation. In addition, we review the literature on alterations of beta-cell mass in T2D and discuss potential techniques to assess beta-cell mass and metabolic defects *in vivo*.

## Introduction

In the year 2030, it is estimated that 439 million people will be affected by diabetes (American Diabetes Association, 2009) and that the number will rise to 642 million by 2040 (Zimmet et al., 2016). Type 2 diabetes (T2D) accounts for 90–95% of all diabetes cases and is a global disease with major health- and financial implications for both the affected and the society. Already, in the 19th century, it was recognized by Lancereaux that there were at least two forms of diabetes which he divided into *diabetes maigre* and *diabetes gras* meaning diabetes of the “thin” and “fat” (National Diabetes Data Group, 1979). With increasing knowledge, the classifications of diabetes have become more detailed and complex, but these early observations still play an important role since they reflect different aspects of pathophysiology. Indeed, diet and body weight have a major impact on the risk of developing T2D which at least in part can explain the dramatic increase in prevalence. Over the last 10 years, there has also been a substantial addition of drugs approved for the treatment of T2D. Despite that, a large number of those affected by T2D fail to reach an acceptable metabolic control (Safai et al., 2018). This can be explained by a number of factors including physical inactivity, diet, adherence to medications but also the underlying pathophysiological process and stage of disease is of importance for the effect of glucose lowering drugs. Over the last years, it has become increasingly recognized that T2D is a heterogeneous disease which requires an individualized treatment with adaptive changes over time as the disease progresses. In addition, hyperglycemia and coupled metabolic defects in diabetes increase the production of oxidative stress and reactive oxygen species (ROS) which can have vast deleterious effects and contribute to beta-cell dysfunction, failure, and loss. As T2D progresses, the initial hyperinsulinemia declines and a large number of patients are rendered insulin deficient due to the loss of beta-cells. In this review, we will highlight the different phenotypical features of T2D and how metabolic defects impact oxidative stress and ROS formation in different tissues. In addition, we review the literature on alterations of beta-cell mass in T2D and discuss potential imaging techniques in order to assess beta-cell mass and metabolic defects *in vivo*.

## The Rationale of Diabetes Classification and Phenotypical Presentations of Type 2 Diabetes

The diagnostic criteria for diabetes mellitus (DM) should preferably be based on parameters and laboratory tests which can be assessed in primary care facilities and broad enough to encompass all afflicted individuals. The more common the disease, the more phenotypically heterogeneous is the affected population. Classification criteria are used to divide the heterogeneous population to more homogeneous subpopulations for research and treatment guidelines (Aggarwal et al., 2015). The current diabetes classification into type 1 diabetes (T1D) and T2D is based on the ability to secrete insulin and the presence or absence of autoantibodies. Patients with T1D must be treated with insulin already at diagnosis whereas patients with T2D initially should be treated with dietary regimes, biguanides, or sulfonylureas. In clinical practice, 90–95% of people that fulfill the DM diagnostic criteria are classified as T2D.

In 1993, Tuomi et al. (1993) identified diabetes patients with a phenotype of both T1D and T2D. Typically, these patients are indistinguishable from T2D at diagnosis but over time they develop a more T1D like phenotype. The subgroup, coined latent autoimmune diabetes in the adult (Brophy et al., 2008), is defined as patients older than 35 years, with glutamate decarboxylase antibodies (GADA) 65 reactive against pancreatic beta-cells and remaining endogenous insulin secretion at least 6 months after diagnosis (Tuomi et al., 1993). The prevalence of LADA ranges from 4–6% in Eastern Asia (Takeda et al., 2002; Zhou et al., 2013) to 10–12% in Northern Europe (Turner et al., 1997; Laugesen et al., 2015), leaving 90% of the heterogeneous T2D population undifferentiated. LADA patients also share genetic characteristics of both T1D and T2D and the rate of beta-cell loss and thereby time to exogenous insulin dependence correlates to the levels of GADA 65 (Niskanen et al., 1995; Fourlanos et al., 2005; Brophy et al., 2008; Cervin et al., 2008). In addition, patients with a classical T1D can develop insulin-resistance despite the lack of endogenous insulin production, often referred to as double-diabetes (Cleland et al., 2013). Thus, the categorization of DM into T1D and T2D is not clear-cut but rather a mix of etiology resulting in a phenotypic continuum (Niskanen et al., 1995; Fourlanos et al., 2005; Buzzetti et al., 2007; Laugesen et al., 2015).

Before 1995, there were only two classes of anti-diabetic agents apart from insulin, sulfonylurea (1946), and biguanide (1959). The identification of LADA implied the need for a tighter glucose monitoring compared to T2D and avoidance of sulfonylurea, but apart from this the classification contributed little to improve the clinical management of diabetes. Since then six pharmacodynamically different drug classes have been approved for clinical use, α-glucosidase inhibitors (1995), thiazolidinediones (1996), metglitinides (1997), glucagon-like peptide-1 (GLP-1) analogs (2005), dipepidylpepidase-4 (DPP-4) inhibitors (2006), and sodium glucose co-transporter 2 (SGLT2) inhibitors (2013) (White, 2014). Aside from providing new treatment possibilities, they elucidate the need for a revised diabetes classification.

From being a rational DM classification in 1979, the classification into T1D and T2D is now obsolete both for research and for clinical guidance. This realization has spurred efforts to find new classifications of diabetes. With a data-driven topologic analysis on electronic medical records data using 73 clinical features associated to variations in single nucleotide polymorphisms, Li et al. (2015) identified three subgroups of T2D that differ both in phenotype and genotype. Subtype 1 (31%) was characterized by obesity, kidney disease, and hyperglycemia whereas subtype 2 (25%) and 3 (44%) were associated with cancer and neurological disease, respectively. The study did not include disease duration and did not reveal if individuals switched subtype over time. However, this study is of interest since it links real-life data to genome-wide association studies.

By using latent class trajectory analysis on a five-time point oral glucose tolerance test (OGTT), Hulman et al. (2018) identified five sub-classes of metabolic control even among non-diabetic individuals that differed in regard to insulin sensitivity and acute insulin response, obesity, lipid levels, and inflammatory markers. The classes were also correlated to different pathophysiological processes. The strongest determinant of time to glucose peak during the OGTT was insulin sensitivity and those patients who shifted sub-class over time could mainly be explained by life-style changes that affect insulin sensitivity.

Ahlqvist et al. (2018) presented a novel diabetes classification by cluster analysis of five phenotypically diabetogenic risk factors. Cluster 1 (severe autoimmune diabetes, SAID) includes all patients with positive GADA 65 antibody titer. Cluster 2 (severe insulin deficient diabetes, SIDD) with low fasting (f) C-Peptide and high HbA1c at diagnosis. Cluster 3 (severe insulin resistant diabetes, SIRD) with high fC-Peptide and high HbA1c. Cluster 4 (mild obesity-related diabetes, MOD) with high BMI and relatively low HbA1c at diagnosis. Cluster 5 (mild age-related diabetes, MARD) was the largest with 39% of the population and characterized by higher age at diagnosis and relatively low HbA1c. During follow-up (median 3.9 years), cluster 2 (SAID) and 3 (SIRD) were more prone to complications than cluster 4 (MOD) and 5 (Abdo et al., 2010).

Udler et al. (2018) aimed to find pathophysiological clusters by takeoff from publically available genome-wide assay study (GWAS). Ninety-four genetic variants and 47 diabetes-related metabolic traits were included to a Bayesian non-negative factorization clustering, which yielded five clusters. The clinical impact of the clusters was then assessed in four separate cohorts (*N* = 17 874). Cluster 1 (beta-cell) and 2 (proinsulin) were associated with beta cell dysfunction, cluster 1 had increased proinsulin levels whereas cluster 2 had decreased proinsulin levels. Clusters 3 (obesity), 4 (lipodystrophy), and 5 (liver/lipid) were associated with mechanisms of insulin resistance. The obesity-liked loci FTO and MC4R were more common in cluster 3, concordantly also waist and hip circumference. Individuals in cluster had decreased adiponectin, low insulin sensitivity index and HDL levels, and increased triglycerides. Cluster 5 was associated with loci related to non-alcoholic liver disease (NAFLD) and these individuals had increased levels of urate and fatty acids related to NAFLD (serum triglycerides, palmitoleic acid, and linolenic acid).

**FIGURE 1:**
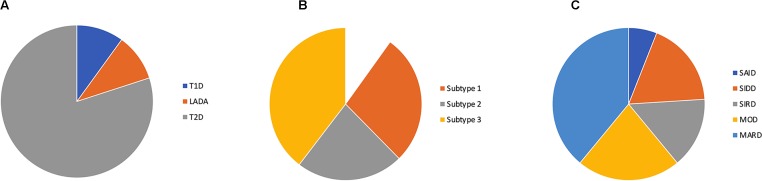
Proportions of diabetes subtypes by **(A)** the current classification, **(B)** subtyping of type 2 diabetes by Li et al. (2015) and **(C)** cluster classification by Ahlqvist et al. (2018) SAID (severe auto-immune diabetes), SIDD (severe insulin deficient diabetes), SIRD (severe insulin resistant diabetes), MOD (mild obestity-related diabetes) and MARD (mild age-related diabetes).

These ambitious attempts to reform diabetes classification, summarized in Figure 1 and Supplementary Table S1, take on the long time insight that diabetes is not a single disease of hyperglycemia, but rather a syndrome of multiple metabolic disturbances. If the addition of genetic and phenotypic parameters actually identifies novel diabetes subgroups, we may well stand in front of a shift of paradigm in both treatment and monitoring diabetes.

## Metabolic Defects and Reactive Oxygen Species in Type 2 Diabetes

Type 2 diabetes, though primarily a disease characterized by decreased insulin sensitivity, also involves the destruction of insulin producing beta-cells during the later stages of the disease (Sakuraba et al., 2002; Butler et al., 2003). An ever-increasing demand for insulin production to overcome progressing insulin resistance becomes harmful to the beta-cells and hyperglycemia and increased free fatty acids, cause oxidative stress (Donath et al., 2005). Albeit the assessment of beta-cell mass in humans has been rather challenging, most current research suggests that beta-cell mass declines with the progression of T2D. While the mechanisms behind beta-cell failure and death in T2D are not fully understood, increasing interest has been directed toward the role of oxidative stress. Increased production of ROS, driven by chronic hyperglycemia and hyperlipidemia, is thought to be a major cause of the beta-cell dysfunction in diabetes (Robertson et al., 2007; Graciano et al., 2013). ROS are known to damage components of the cellular machinery, including DNA, proteins, and lipids which leads to a vast array of deleterious effects (Schieber and Chandel, 2014). In fact, signs of increased ROS activity have been observed in pancreatic islets of deceased T2D patients (Sakuraba et al., 2002). Moreover, oxidative stress likely contributes to the development of peripheral insulin resistance and many of the long-term micro- and macrovascular complications of diabetes (Styskal et al., 2012).

Reactive oxygen species are inevitable byproducts of aerobic metabolism, produced primarily from “leakage” of electrons in the mitochondrial electron transport chain. Under basal conditions, ROS serve in various pathways regulating biological and physiological processes involving mainly stress response signaling (Schieber and Chandel, 2014). Antioxidant enzymes and low molecular ROS-scavengers balance ROS activity in the physiological redox biology. An overexpression of ROS, or overwhelming of the antioxidant responses, results in oxidative stress. Accumulating evidence suggests that oxidative stress is involved both in the early events surrounding the development of T2D, as well as the later hyperglycemia induced tissue damage (Nowotny et al., 2015). Moreover, pancreatic islets and particularly beta-cells express less antioxidant enzymes compared to other tissues, making them more susceptible to the damaging effects of oxidative stress and ROS (Lenzen et al., 1996; Miki et al., 2018).

While excessive ROS levels have long been viewed as responsible for many undesirable effects (Stadtman, 1992, 2001), it is becoming more evident that moderate levels of ROS are often not only inevitable but also necessary and beneficial for many normal cellular functions (Cai and Yan, 2013), this is the case also in beta-cells. In the production of insulin, ROS are unavoidable byproducts of enzyme driven (Sevier and Kaiser, 2002; Tu and Weissman, 2004) folding of proinsulin in the endoplasmic reticulum (ER). With each formation of a disulphide bond, one molecule of ROS is produced. With the insulin molecule having three disulphide bonds important for its function (Chang et al., 2003), production of one molecule of insulin would be associated with the production of three molecules of ROS. Hyperglycemic conditions can cause a 50-fold increase in insulin biosynthesis (Goodge and Hutton, 2000). Under these conditions, beta-cells each produce up to 1 million molecules of insulin per minute (Scheuner and Kaufman, 2008), this could possibly signify production of 3 million molecules of ROS per minute in every beta-cell (Gross et al., 2006; Shimizu and Hendershot, 2009). Regulation of insulin translation and the unfolded protein response (UPR) play an important role, but the exact mechanisms by which beta-cells cope with this amount of ROS production are still not fully understood, particularly as they express relatively low levels of antioxidant enzymes. Autophagy also protects against oxidative stress and ER-stress (Kroemer et al., 2010), and failure of this system may worsen beta-cell function in diabetic conditions (Watada and Fujitani, 2015).

Shimizu and Hendershot (2009) elegantly summarize possible molecular mechanisms coping with oxidative stress in secretory tissues. Common for the protective mechanisms, such as the UPR, is that prolonged activation often results in diversion to cytodestructive effects, giving us a possible explanation of how ROS might take part in the beta cell loss observed particularly in T1D. If conditions of ER-stress are not resolved, Ca2+ leaks from the ER, further increase ROS production by causing mitochondrial dysfunction (Deniaud et al., 2008), thus leading to an increased oxidative stress load in the already struggling cells, and subsequently apoptosis. This ties the role of ER-stress and oxidative stress in with the notion that mitochondrial apoptotic pathway plays a central role in cytokine induced beta-cell failure in T1D (Grunnet et al., 2009). Furthermore, ER-stress in beta-cells has also been suggested to be in part responsible for sustaining the autoimmune response observed in T1D (Marré et al., 2015).

It is also conceivable that during diabetic conditions, when demand for insulin production is increased, and beta-cell ROS production remains high for prolonged time periods, further insulin production is inhibited as a cytoprotective measure. Indeed, Ire1, which is one of the effector branches of the UPR, when continuously activated has been shown to cause suppression of insulin gene expression (Lipson et al., 2006), possibly explaining why prolonged hyperglycemia in T2D patients leads to diminished insulin production also in the absence of apoptosis (Shimizu and Hendershot, 2009). Thus, the subject of ROS is both complex and rather paradoxical, being both an integral part of the islets basic functioning, and perhaps even necessary for their proliferation, but left uncontrolled part of their demise.

In addition to ROS production from increased metabolism and insulin production, hyperactivity in the NADPH oxidases (NOX) also leads to excessive ROS production. Seven membrane bound isoforms of the NOX enzymes (NOX1-5 and DUOX1-2) have been identified. These perform normal cellular functions at physiological conditions, but excessive activation produces harmful levels of ROS. Increased activity of some NOX isoforms has been shown to play an important role in metabolic defects and diabetes through mitochondrial dysregulation in the beta-cells (Guichard et al., 2008; Syed et al., 2011). Increased NOX activity has also been linked to lipid induced ROS production and fatty acid promoted amplification of glucose-stimulated insulin secretion (Graciano et al., 2013). Some NOX isoforms seem to be activated by glucose stimulation, and in the short term potentiate insulin release (Morgan et al., 2009); however, excessive long-term activation is detrimental to beta-cell function (Syed et al., 2011). The NOX4 isoform has been suggested to function as a mitochondrial energy sensor, being negatively regulated by ATP (Shanmugasundaram et al., 2017), and might thus be a source of ROS in both islets and other tissues experiencing metabolic stress in diabetes. Of great interest, we have recently demonstrated that selective NOX4 inhibitors protect human islets and reduce beta-cell death under *in vitro* conditions mimicking the T2D environment (Wang et al., 2018a), making it a potential drug target.

Advanced glycation end products (AGEs) are modified proteins and lipids formed under conditions of oxidative stress. AGEs can, however, also sustain oxidative stress by increasing ROS formation and negatively impact antioxidant systems (Nowotny et al., 2015). Moreover, AGEs such as methylglyoxal are highly abundant in the standard western diet (Uribarri et al., 2007), rendering them as both endogenous and exogenous contributors to oxidative stress. While the full role of AGEs in T2D is not yet completely understood, it is generally accepted that they play an important role by contributing to the oxidative stress, causing both beta-cell damage and peripheral insulin resistance (Vlassara and Uribarri, 2014).

Besides oxidative stress related to glucolipotoxicity, AGEs, and dietary factors, there are many other environmental factors associated with deterioration of beta-cell function that are less well understood. One such factor is disruption of the islets circadian rhythm, which has also recently been suggested to cause increased ROS production and a decreased production of anti-oxidant genes in beta-cells, leading to beta-cell dysfunction and diabetes (Lee et al., 2018).

Numerous studies have attempted to delineate the potential antioxidant effects of current oral antidiabetic treatments as well as for exogenous insulin substitution, with biguanides being the most studied substance. The Biguanide Metformin has been evaluated as a potential treatment for many diseases apart from T2D with promising results. Its potential use in various forms of cancer (Cheng and Lanza-Jacoby, 2015; Vancura et al., 2018), infectious diseases (Kajiwara et al., 2018), cardiovascular disease (Diaz-Morales et al., 2017; Nesti and Natali, 2017), skin disorders (Wang et al., 2018b), and much more is continuously being investigated. In many cases, alterations in redox status are suggested as a main mechanism of action. While Metformin is suggested to ameliorate many disorders by decreasing oxidative stress (Cheng and Lanza-Jacoby, 2015; Diaz-Morales et al., 2017), others suggest the opposite, that Metformin acts by increasing ROS production (Kajiwara et al., 2018; Wang et al., 2018b). While at first glance these conflicting reports seem discerning, it is not unexpected that cells and tissues with vastly differing physiological processes respond differently to Metformin and oxidative stress. In general, research concerning the effects of Metformin on oxidative stress in diabetes suggests that it decreases peripheral ROS production and thereby protects against diabetic atherosclerosis and other complications (Esteghamati et al., 2013; Singh et al., 2016; Diaz-Morales et al., 2017). A recent study implies that aberrant complex I activation in the pancreas of diabetic patients causes an overflow of NADH that is diverted into ROS production leading to beta-cell dysfunction and death (Wu et al., 2017), and Metformin being a mitochondrial complex I inhibitor effectively counteracts this. So, while Metformin’s main mechanism of action in reducing hepatic gluconeogenesis and increasing peripheral insulin sensitivity has been known for years, many of its functions are yet to be fully understood (Rena et al., 2017), including its effect on oxidative stress.

Multiple studies also suggest that GLP-1 analogs may positively influence redox homeostasis, summarized in Petersen et al. (2016). When it comes to other oral antidiabetics, some research suggests effects on ROS production in various tissues by sulfonylureas (Sawada et al., 2008), α-glucosidase inhibitors (Aoki et al., 2012), thiazolidinediones (Singh et al., 2016), and DPP4 inhibitors (Rizzo et al., 2012), but it must be noted that research on these matters are limited.

In a study where exogenous insulin analogs were administered to T2D patients who failed to achieve satisfactory glycemic control on Metformin and sulfonylurea alone, showed a significant decrease in oxidative stress markers (Tuzcu et al., 2013). Interestingly, this was not related to changes in mean glucose levels, suggesting instead some direct inhibitory effects on ROS formation. While insulin in high levels may promote oxidative stress (Rains and Jain, 2011), this study and others (Monnier et al., 2011) suggest that insulin has a rather complex relationship with oxidative stress in T2D.

The relationship between glucose variability and oxidative stress in T2D has also been examined and has yielded somewhat conflicting results. Monnier et al. (2011) reported a significant correlation between glucose variability and oxidative stress whereas a repeating study failed to find a relationship (Siegelaar et al., 2011). A possible explanation for this, as the authors mention in their discussion, is that the latter study mainly examined patients with significantly better glycemic control. This hypothesis is strengthened by results from another study by Monnier et al. (2010) where the relationship between glucose variability and oxidative stress was also associated with HbA1c.

A major limitation for studies of oxidative stress *in vivo* is the difficulty to measure ROS in a reliable way, summarized by Halliwell and Whiteman (2004). In general, the short half-life of reactive species limits our possibilities to measure them directly. Instead, we are limited to measuring the levels of markers for oxidative damage or trapping the reactive species and measuring levels of the trapped molecules. This entails a number of problems, as the marker or trap preferably has to be stable, specific, quantifiable, present in the studied tissue and in addition not confounded by diet or alternative activation pathways. As of now, there are no biomarkers of ROS that are considered to be ideal, but some are better than others. For instance, isoprostanes are considered a rather reliable biomarker for lipid peroxidation, which is a common way to measure the effects of oxidative stress *in vivo* (Halliwell and Whiteman, 2004; Kaviarasan et al., 2009). Indeed, the levels of isoprostanes have been found to be increased in T2D (Kaviarasan et al., 2009).

**FIGURE 2:**
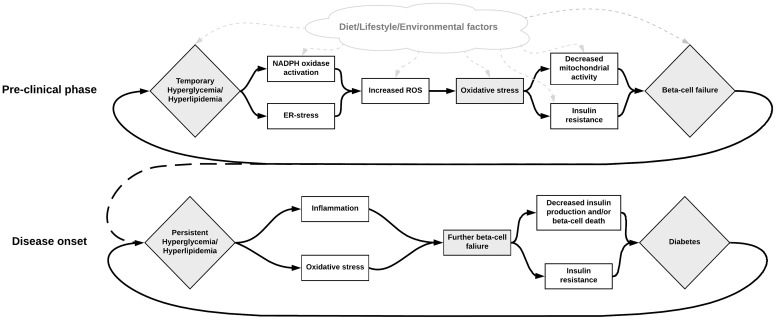
Illustration of the possible role of NOX activation in the development of beta-cell failure, hyperglycemia, and diabetes. Metabolic dysregulation leading to hyperactivity in the NOX-enzymes results in excessive ROS production and oxidative stress. This increased oxidative stress may subsequently be responsible for beta-cell failure, which in turn contributes to increased metabolic dysregulation. Various other factors may also influence these steps in different ways. For instance, diet can contribute to increased oxidative stress directly by containing excessive AGEs, or indirectly by contributing to the metabolic dysregulation. Inhibition of the NOX enzymes seems to be a promising solution for breaking this deleterious cycle.

In conclusion, there are multiple pathways for excessive ROS formation in T2D, and a deficit in the beta-cells capacity to cope with oxidative stress, summarized in Figure 2. Oxidative stress may not only be caused by a number of metabolic defects in T2D but can also in itself contribute to aggravating the defects and the different phenotypes of diabetes. While there exist some difficulties in studying oxidative stress *in vivo* in humans, our current understanding is that it appears to have a central role in many processes involving the development and progression T2D and its long-term complications. Aside from assessing redox properties of currently available medication, novel treatments targeting ROS production such as specific NOX inhibitors are also being researched (Wang et al., 2018a).

## Adaptive Changes of Beta-Cell Mass in Obesity and Type 2 Diabetes

Due to the lack of established *in vivo* techniques, our current knowledge on beta-cell mass in humans fully relies on autopsy studies. Although a very valuable source, autopsy material has a number of drawbacks including technical difficulties but of outmost importance is the inherent lack of repeated measurements. The number and volume of islets increase substantially from fetal life to adulthood and is estimated to increase fivefold from birth to adulthood, in parallel the exocrine pancreas increase 15-fold in size (Witte et al., 1984). Therefore, islets compose 20% of the total pancreas volume in newborns, 7.5% in children but only 1–2% in adults (Witte et al., 1984). In addition, the proportion of beta-cells within the islets varies in the different anatomical regions of the pancreas with more beta-cells in corpus and cauda. In adults, the pancreas weighs in average around 100 g but can range from 50 to approximately 170 g. Combined with the potential twofold difference in islet percentage, this gives a ≥fivefold theoretical difference in islet mass even among healthy individuals. Well in line with this, a fivefold difference in beta-cell mass is often observed among healthy individuals in reports based on autopsy material (Rahier et al., 2008). This is important to keep in mind since our knowledge and view on beta-cell mass in T2D is based on comparisons with healthy controls.

In adulthood there are in particular two physiological conditions which lead to an increase in beta-cell mass; pregnancy and obesity (Van Assche et al., 1978; Kloppel et al., 1985; Butler et al., 2010; Hanley et al., 2010; Saisho et al., 2013). The alterations of beta-cell mass during pregnancy are elegantly reviewed by Nielsen (2016). Of importance, beta-cell mass has been found to be increased by 50% in obese individuals when compared to lean individuals (Rahier et al., 1983; Kloppel et al., 1985; Hanley et al., 2010; Saisho et al., 2013). In fact, Saisho et al. (2013) found that beta-cell mass correlates with BMI. In patients with T2D, there have been conflicting results on beta-cell mass which can be explained by the above-mentioned difficulties regarding comparisons with non-diabetic individuals and by the disease duration and heterogeneity of T2D. In patients with recent onset T2D, the beta-cell mass has been found to be unaltered (Rahier et al., 1983; Hanley et al., 2010); however, in the report by Hanley et al. (2010) obese patients with T2D displayed a decreased beta-cell mass. The unaltered beta-cell mass in recent onset T2D in combination with the normal or increased C-peptide levels make a strong argument for that T2D is not primarily developed due to a loss of beta-cells but rather due to insulin resistance and beta-cell dysfunction. Others have reported on a moderate (≈25%) decrease in beta-cell mass (Sakuraba et al., 2002) but there are also reports on a more pronounced reduction (≈50%) of beta-cell mass in patients with long-standing T2D (Maclean and Ogilvie, 1955; Butler et al., 2003; Rahier et al., 2008). The degree of beta-cell loss in T2D has been found to correlate to disease duration, with a distinct reduction after 20 years of disease (Rahier et al., 2008). In addition, there are a number of reports supporting the deposition of amyloid in islets of patients with T2D which could contribute to beta-cell dysfunction and apoptosis (Bell, 1952; Ehrlich and Ratner, 1961; Clark et al., 1988; Sakuraba et al., 2002; Huang et al., 2007). However, the role of islet amyloid deposits in the pathogenesis of T2D is beyond the scope of this review, but for the interested the comprehensive review on islet amyloid by Westermark et al. (2011) is warmly recommended.

In non-diabetic individuals, the beta-cell mass is tightly regulated during adulthood by a balance between beta-cell replication and apoptosis (Bonner-Weir, 2000). Interestingly, ROS is known to increase apoptosis and in high concentrations induce cell cycle arrest. However, in low to moderate concentrations, ROS have been found to stimulate cell proliferation (Boonstra and Post, 2004; Ahmed Alfar et al., 2017). In addition, experimental studies have shown that mitochondrial ROS play an important role in beta-cell proliferation (Ahmed Alfar et al., 2017) and in the establishment of beta-cell mass during development (Zeng et al., 2017). The loss of beta-cell mass in T2D can be explained by the increased apoptosis rate observed in islets from T2D patients (Butler et al., 2003; Rahier et al., 2008; Hanley et al., 2010) and the lack of increased beta-cell proliferation (Butler et al., 2003). Of great interest, pancreatic beta-cells inherently express low levels of antioxidant enzyme superoxide dismutase (Falk-Delgado et al., 2015) but there is also support of further decreased levels of SOD in beta-cells of T2D patients (Sakuraba et al., 2002). This further supports the role of beta-cell failure and loss due to ROS activity in T2D.

The combined evidence suggests that beta-cell mass is relatively unaltered at the onset of T2D, but declines progressively with the disease. It seems as if the loss of beta-cell mass is more pronounced in obese T2D individuals which could be due to a loss of stimulatory signals or a lack of beta-cell proliferatory response to obesity in combination with an increased metabolic stress under diabetic conditions. In addition, the local milieu and metabolic challenges of beta-cells in T2D obesity may also have deleterious effects (Prentki et al., 2002) and increase the production of ROS (Graciano et al., 2013).

## *In vivo* Imaging of Beta-Cell Mass

Given the pandemic increase in T2D and the heterogeneous nature of the disease in combination with large individual variations in beta-cell mass, it would be of great importance to establish a technique allowing *in vivo* monitoring of beta-cell mass. With such a technique, we would gain valuable insight on the pathophysiology of the disease and the decline of beta-cell mass over time in different phenotypical presentations of T2D. Due to the size and distribution of pancreatic islets, imaging beta-cell mass is a tough challenge since there are no non-invasive imaging modalities with a high enough resolution to delineate single islets in human. However, by using positron emission tomography (PET) in combination with a beta-cell specific PET-tracer, it would be possible to monitor the combined signal from all islets within the pancreas and thereby beta-cell mass. There have been several attempts to find a beta-cell specific PET-tracer over the last decade, summarized in a recent review by Eriksson et al. (2016).

We have focused our attempts on the clinically available PET-tracer [^11^C]5-hydroxy-tryptophan ([^11^C]5-HTP), a serotonin precursor, which, however, is not completely specific for beta-cells but is also retained in remaining endocrine cells within the islets of Langerhans. Using PET in combination with computed tomography (CT), we have found that the pancreatic uptake of [^11^C]5-HTP is reduced by 66% in patients with long-standing T1D when compared with healthy controls, which is well in line with a complete beta-cell loss (Eriksson et al., 2014). We have also evaluated the use of [^11^C]5-HTP in combination with magnetic resonance tomography (MRT) in patients with T2D. Patients were first categorized into four groups based on BMI (lean or obese) and treatment regime, either oral antidiabetic drugs (OADs) alone or in combination with insulin (OAD+insulin). The functional beta-cell mass was determined based on acute C-peptide response to a bolus of arginine and C-peptide response to a glucose potentiated arginine test. We found that the patients, both lean and obese, treated with OAD had a normal C-peptide response to arginine but a marked reduction of C-peptide secretion in response to the glucose potentiated arginine test. Patients treated with OAD+insulin displayed a marked reduction in C-peptide secretion in both the acute- and glucose potentiated arginine test. However, the pancreatic uptake of [^11^C]5-HTP did not differ between the groups (Carlbom et al., 2017). Of interest, we observed a twofold difference of [^11^C]5-HTP pancreatic uptake in healthy controls but among the T2D group the difference was close to fivefold. In fact, among lean T2D patients with OAD+Insulin two thirds of the patients displayed a pancreatic uptake well below the absolute levels of healthy controls. In line with this, the pancreatic volume was quite homogenous in healthy controls but varied in T2D groups with a significantly increased volume in obese T2D patients with OAD+insulin.

There could be several contributors to the observed discrepancy between functional beta-cell mass and [^11^C]5-HTP pancreatic uptake. In fact, isolated islets from patients with T2D contain and secret less insulin when compared to non-diabetic donors (Deng et al., 2004; Marchetti et al., 2004). Since [^11^C]5-HTP is not a beta-cell specific tracer, the result could still reflect a true finding of islet mass in T2D since the alpha-cell mass has been found to be unaltered (Henquin and Rahier, 2011) and there have been reports supporting a de-differentiation of beta-cells rather than a beta-cell destruction in T2D (Talchai et al., 2012; Spijker et al., 2015). However, the data should be interpreted with caution given the small number of individuals in each group. Given the large variability even in normal physiology, cross-sectional comparisons will be difficult in order to discern more discrete alterations of beta-cell mass. However, this could be overcome by the use of repeated paired measurements. Indeed, by using a retrospective design of repeated examinations, we have found that the pancreatic uptake of [^11^C]5-HTP in T2D decrease over time as the disease progresses (Eriksson et al., 2014). In addition, new PET-traces targeting GPR44 that are specific for beta-cells are currently being developed which could potentiate future studies of adaptive changes of beta-cell mass during different stages of T2D (Eriksson et al., 2018).

## *In vivo* Imaging of Glucose- and Lipid Metabolism in Type 2 Diabetes

Apart from the possibilities of imaging and quantifying beta-cell mass with PET, the technique also opens up for possibilities of mapping metabolic alterations in specific tissues *in vivo*. As discussed above, hyperglycemia and hyperlipidemia increase ROS activity which contributes to tissue damage and diabetic complications. Currently, we base most of our clinical decisions and classifications on the circulating levels of glucose, free fatty acids, and hormones. We can also detect indirect measurements of ROS but it has been difficult to establish reliable biomarkers, in addition the effects and activity of ROS can vary in different tissue. However, by using PET, we could actually image and map how these metabolic defects occur in different tissues which could relate to the risk of complications in T2D. In addition, these techniques would also provide important insights on the pathophysiological processes and thereby guide us in which treatments to use. An important advantage with PET is the relatively low radiation burden associated with tracers and the possibility to use MRT for anatomical mapping. In combination with the short radioactivity half-life of most tracers (<120 min), it is therefore possible to perform repeated examinations and to use a multi-tracer approach which makes it possible to examine several metabolic pathways at the same time.

The most widely used PET-tracer is in fact a glucose analog, 2-deoxy-2-(^18^F)fluoro-D-glucose ([^18^F]FDG), which is used in different fields of medicine to identify everything from tumors to inflammation. [^18^F]FDG is a general biomarker for any tissue relying on glycolysis, for example, the brain and the myocardium. Briefly, by relating the uptake rate of [^18^F]FDG in different tissues to the glucose levels in plasma, a metabolic rate of glucose (MRGlu, μmol/g/min) in specific tissue can be determined (Phelps et al., 1979). By using [^18^F]FDG PET, it has, for instance, been demonstrated that the brain glucose utilization is not affected by insulin-infusion in healthy individuals, i.e., the glucose uptake is maximized already at fasting conditions. However, in individuals with impaired fasting glucose (IGF), the brain glucose utilization increases in response to insulin infusion suggesting that the brain glucose metabolism is disturbed even in IFG (Hirvonen et al., 2011). Using whole-body [^18^F]FDG PET and hyperinsulinemic euglycemic clamp, it was recently demonstrated that the brain glucose utilization is increased in patients with T2D (Boersma et al., 2018). In contrast, the authors found that the glucose utilization was decreased in skeletal muscle, visceral- and adipose tissue, and the liver in patients with T2D (Boersma et al., 2018).

In addition, several fatty acids have been labeled with positron emitting nuclides in order to track their fate in the human body (Mather and DeGrado, 2016). Fatty acid uptake and the rate of oxidation can be determined by relating the uptake and retention, respectively, of the labeled fatty acid with the circulating amounts of non-esterified fatty acids in plasma (p-NEFA). Some of the clinically more commonly used markers for fatty acid metabolism are [^11^C]Palmitate (Weiss et al., 1976) and 14-^18^F-fluoro-6-thia-heptadecanoic acid ([^18^F]FTHA) (DeGrado et al., 1991). Depending on the design of the PET tracers and the trapping mechanisms in different cellular compartments, the tracers tend to reflect either fatty acid uptake or fatty acid oxidation. By using a multi-tracer approach, it has been demonstrated in patients with T2D that the myocardial metabolism is shifted toward fatty acid oxidation instead of glycolysis (Rijzewijk et al., 2009). In addition, it has been demonstrated by PET imaging that patients with T2D have an increased myocardial fatty-acid uptake and fatty-acid oxidation compared to healthy individuals (Mather et al., 2016). In both of these studies, the patients displayed a good metabolic control and had not yet established any micro- or macrovascular complications.

As discussed, metabolic defects increase production of ROS and by imaging metabolism in different tissue using PET this could give indirect evidence on ROS formation and activity. In fact, the uptake of [^18^F]FDG has been linked to ROS concentration in tumor cell-lines and tumor-bearing mice (Jung et al., 2013). Also, increasing oxidative stress has been related to decreased brain [^18^F]FDG uptake in neurodegenerative disorders (Mosconi et al., 2008). However, the volatile nature of ROS in tissue results in a less than exact assessment using indirect approaches for measurement. Therefore, it is potentially a major advancement that PET tracers specific for ROS are now being developed. In experimental studies, [^18^F] and [^11^C] labeled dihydrophenantridine derivatives have been used which bind to DNA in their oxidized forms and therefore becomes trapped within cells. The uptake was found to be ROS specific in both *in vitro* and *in vivo* experimental studies (Chu et al., 2014; Wilson et al., 2017). In addition, also other imaging techniques are being developed for measuring free radicals directly in living organisms by using electron spin resonance and special probes (Berliner et al., 2001; Elas et al., 2012). However, these probes are currently only available for preclinical use. Furthermore, genetically encoded ROS probes such as HyPer-3 (Bilan et al., 2013) and roGFP2-Orp1 (Gutscher et al., 2009) have been presented in recent years (Shimizu and Hendershot, 2009; Meyer and Dick, 2010), and while perhaps not suited for the clinic, allows visualization of specific ROS detection *in vivo* in disease models. PET imaging using tracers targeting ROS will likely soon be available in the clinical setting, which will be a valuable contribution in many fields of medicine, not the least in diabetes research.

## Discussion

Given the diverse nature of T2D, it is a challenging and costly disease to manage, both in perspective of the individual patient, as well as the society as a whole. Classification and monitoring of diabetes has until recently relied primarily the on quantification of circulating glucose and insulin levels in combination with the presence or absence of autoantibodies. While this is a massive step up from the characterizations of Lancereauxs classification based on body weight, it falls short for use in modern research and drug development. Common for the classifications by Li et al. (2015), Ahlqvist et al. (2018), and Udler et al. (2018) is that they see T2D as a result of different pathophysiological disturbances and that the heterogeneity can be explained by identifying which disturbance is the dominant. Li et al. (2015) and Udler et al. (2018) emphasize genetic variance as the underlying cause and originate their classification from there. They find expected concordance between genetic and phenotypic traits in several cohorts. Ahlqvist et al. (2018) and Hulman et al. (2018) take on a more pragmatic approach by focusing on phenotype characteristics. By using variables that can be easily measured in primary care, the classification by Ahlqvist et al. (2018) has the potential to be widely accepted. However, the use of phenotype for classification may not be robust over time. Successful treatment may lead to a switch in cluster belonging, requiring re-classification on a regular basis.

Type 2 diabetes is associated with a number of metabolic defects resulting from decreased insulin sensitivity, many of which likely take part in the development and progression of the disease. Accumulating evidence points toward oxidative stress as a culprit, responsible not only for the devastating consequences of peripheral hyperglycemia and hyperlipidemia, but also for the dysfunction and loss of beta-cells. Multiple sources for this oxidative stress have been suggested (Styskal et al., 2012; Graciano et al., 2013; Nowotny et al., 2015). Much points to insulin deficiency being correlated with increased oxidative stress, providing a possible explanation to why the SIDD and SIRD classes suggested by Ahlqvist et al. (2018) are more prone to complications than MOD and MARD during follow-up. As elevated insulin levels promote oxidative stress, the insulin resistance observed in SIRD may in part be caused by elevated peripheral oxidative stress. SIDD in contrast, characterized by insulin deficiency, while having similar peripheral complications as SIRD, may initially have its oxidative stress primarily localized to the pancreatic islets, causing impairment of beta-cell function and survival. Improved understating of the metabolic defects that occur, the implications of oxidative stress, and delineation of mechanisms which are most important for the progression of the disease, will help us combat T2D as well as provide potential targets for novel treatment strategies. In order to succeed in this challenging task, both basic research and clinical studies are warranted to pinpoint the exact mechanisms. For the latter, effective and specific monitoring tools are needed. With the pancreas being a quite inaccessible organ for invasive *in vivo* studies, the assessment of inflammation, beta-cell mass, and metabolism have so far been mostly been limited to autopsy studies. Furthermore, adversities in studying the mechanisms behind and processes surrounding beta-cell failure and death in T2D, especially *in vivo*, are likely present not solely due to limitations in methodology, but also because of de facto differences in mechanisms between phenotypically different subgroups. The ongoing development of non-invasive PET imaging techniques targeting beta-cell mass, as well as glucose and lipid metabolism and ROS, will hopefully provide us with tools to perform more extensive prospective studies in order to delineate the pathophysiological changes in the progression of diabetes. In addition, this will serve as a valuable tool for evaluating the effects of novel drug interventions and may also aid in further sub-classification attempts in order to tailor specific treatment regimes. Much of our current knowledge support the view that the loss of beta-cell mass is not the cause of but rather the effect of T2D. However, a number of important questions regarding the role of beta-cell loss in T2D remain unanswered. With the development of *in vivo* techniques based on PET for the assessment of beta-cell mass, a number of important questions can be addressed.

## Conclusion

Type 2 diabetes is a complex and heterogeneous disease which affects millions of people in increasing numbers worldwide. T2D increases the risk of cardiovascular disease and causes a number of long-term complications with dramatic effects for both the affected individual and the society. The classification of diabetes is at an interesting turning point and we will likely have a number of sub-classifications of T2D within the next few years. Some of the metabolic defects of T2D are causal for the disease but many are secondary and can further contribute to an aggravated metabolic control, beta-cell dysfunction, and even beta-cell loss. An important, and perhaps so far underestimated, factor is the role of oxidative stress and ROS in the pathophysiology of T2D. ROS could be causal but also contribute to a large number of the metabolic defects observed in T2D including beta-cell dysfunction and beta-cell loss. Beta-cell mass is unaltered at the onset of T2D but progressively declines over time. Currently, our knowledge on beta-cell mass is limited to autopsy studies and based on comparisons with healthy controls. PET in combination with novel PET-tracers holds great potential for quantifying beta-cell mass *in vivo*. In addition, PET can be used to quantify and image metabolic defects as well as ROS activity in different tissues. With the use of these novel techniques, we anticipate that our understanding on the pathophysiology of T2D will dramatically increase over the coming years which hopefully will result in the development of new potent drugs to combat metabolic defects, ROS activity and beta-cell failure in T2D.

## Data Availability Statement

The datasets for this manuscript are not publicly available because historical published data will be provided by the corresponding author upon request. Requests to access the datasets should be directed to daniel.espes@mcb.uu.se.

## Author Contributions

DE and AE are responsible for the design of the manuscript. All authors have contributed in the writing and critical review of the manuscript.

## Conflict of Interest Statement

The authors declare that the research was conducted in the absence of any commercial or financial relationships that could be construed as a potential conflict of interest.
